# Supplementary figures and images for: Achieving Influenza Vaccine Uptake Target in Canada via a Pharmacy-Led Telephone Discussion during the 2019–2020 Season
Source: Vaccines (Basel). 2021 Mar 26;9(4):312. doi: 10.3390/vaccines9040312 (PMC8065524; doi:10.3390/vaccines9040312)

Supplementary File A – Strategy and flow chart of the pharmacist intervention

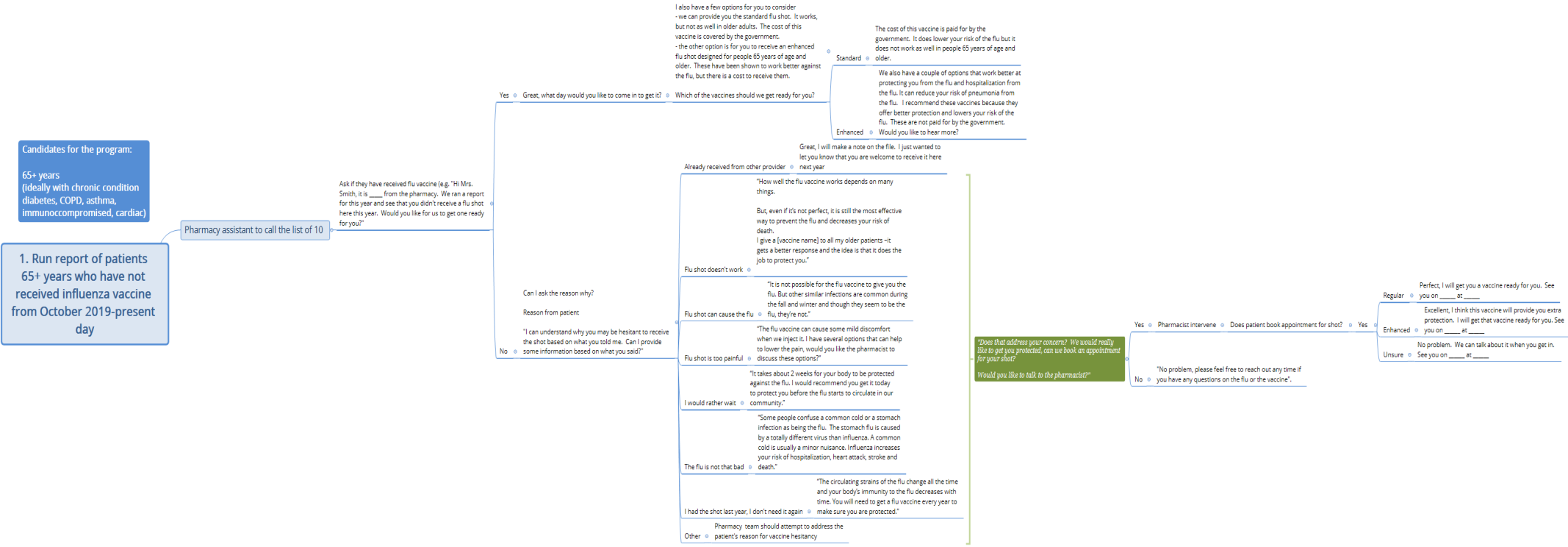

Supplement: Supplementary file 1 [file vaccines-09-00312-s001.pdf]
